# Effects of H2-Receptor Antagonists on the Exposure of Dacomitinib

**DOI:** 10.3390/pharmaceutics16010118

**Published:** 2024-01-17

**Authors:** Jian Liu, Swan Lin, Anthony Huynh, Weiwei Tan

**Affiliations:** 1 Clinical Pharmacology, Pfizer Investment Co., Ltd., Beijing 100010, China; jian.liu2@pfizer.com; 2 Clinical Pharmacology, Global Product Development, Pfizer Inc., San Diego, CA 92121, USA; swandlin@gmail.com; 3 Skaggs School of Pharmacy and Pharmaceutical Sciences, University of California, San Diego, CA 92093, USA

**Keywords:** dacomitinib, EGFR inhibitor, overall survival, pharmacokinetics, progression-free survival, proton-pump inhibitors

## Abstract

Dacomitinib is an irreversible epidermal growth factor receptor (EGFR) tyrosine kinase inhibitor indicated for the treatment of patients with advanced non-small-cell lung cancer (NSCLC) and EGFR-activating mutations. Proton-pump inhibitors decreased dacomitinib exposure. This analysis summarizes the effect of Histamine-2 receptor antagonists (H2RAs) on dacomitinib exposure. A within-patient comparison of the steady-state trough concentrations (C_trough,ss_) of dacomitinib and its active metabolite and active moiety with and without concomitant use of H2RAs was conducted using a linear mixed effects model with pooled data from 11 clinical studies in patients with NSCLC. An oral absorption physiologically based pharmacokinetic (PBPK) model was constructed and verified using clinical pharmacokinetic (PK) data after a single dose of dacomitinib in healthy volunteers to estimate the effect of gastric pH altered by an H2RA on dacomitinib’s PKs. The adjusted geometric mean of the dacomitinib C_trough,ss_ of the dacomitinib parent, metabolite and active moiety following co-administration with an H2RA was approximately 86%, 104% and 100% relative to that following dacomitinib 45 mg administration without an H2RA (*p* > 0.05). The PBPK modeling showed negligible change in dacomitinib maximum concentration (C_max_) and area under the drug concentration–time curve (AUC) over 0–24 h after H2RA administration when compared with those administered dacomitinib alone. Co-administration of an H2RA with dacomitinib is not expected to have any clinically relevant effect on dacomitinib exposure.

## 1. Introduction

Dacomitinib is an irreversible adenosine triphosphate (ATP)-competitive, orally bioavailable, small-molecule inhibitor of the ErbB human epidermal growth factor receptor (HER) family of receptor tyrosine kinases (RTKs), including the epidermal growth factor receptor (EGFR) [1,2]. In a randomized, open-label, phase 3 trial (ARCHER 1050), dacomitinib statistically significantly improved progression-free survival and overall survival versus gefitinib, a first-generation EGFR tyrosine kinase inhibitor, as a first-line treatment in patients with EGFR mutation-positive non-small-cell lung cancer (NSCLC) [3,4,5]. On the basis of the results from ARCHER 1050, dacomitinib was approved for the first-line treatment of patients with metastatic NSCLC containing EGFR exon 19 deletion or exon 21 L858R substitution in the United States and the European Union [2,6].

Cancer patients frequently take acid-reducing agents (ARAs), such as antacids, histamine H2-receptor antagonists (H2RAs), and proton-pump inhibitors (PPIs), to alleviate symptoms of gastroesophageal disease, thereby raising the potential for drug–drug interactions (DDIs) that could decrease the exposure of anticancer medication and result in subsequent compromised therapeutic outcome. Many approved, orally administered, small-molecule tyrosine kinase inhibitor (TKI) drugs for cancer treatment are weak bases that exhibit pH-dependent solubility [7]. The increase in gastric pH may limit the absorption of TKI drugs that require an acidic environment for optimal dissolution, which in turn can lead to decreased plasma exposure [8]. Consequently, the oral bioavailability of these drugs may be significantly influenced when co-administered with ARAs.

The aqueous solubility of dacomitinib is pH dependent, with the highest solubility observed at acidic pH [9]. A few of clinical studies have been conducted to assess whether the absorption of dacomitinib may be affected by ARAs that increase stomach pH [9,10,11]. Results from a clinical study in healthy volunteers showed 7 days of continuous dosing with rabeprazole 40 mg, a PPI, reduced dacomitinib C_max_ and AUC0-96 by 51% and 39%, respectively, following a single, 45 mg dose of dacomitinib [9]. Furthermore, T_max_ was delayed from 5 to 6 h (dacomitinib alone) to 12 h (dacomitinib co-administered with PPI) [9]. On the other hand, treatment with 20 mL of locally acting antacid (Maalox^®^ Maximum Strength, 400 mg/5 mL) did not cause clinically relevant changes in dacomitinib concentrations [2]. Based on these findings, it is recommended that concomitant use of PPIs with dacomitinib should be avoided, and locally acting antacids can be taken instead [2]. H2RAs are ARAs which competitively block histamine H2 receptors and interfere with one of three pathways for proton-pump activation, resulting in a substantial reduction in acid secretion, but are less potent than PPIs. The effect of Histamine-2 receptor antagonists (H2RAs) on dacomitinib exposure has not been studied in a dedicated clinical pharmacology study. Based on physiology and knowledge from other molecular targeted agents with pH-dependent absorption, a staggered dosing approach (administration of dacomitinib at least 2 h before or 10 h after H2RA administration) is recommended on the dacomitinib label [2,6] in order to allow dacomitinib absorption at the time of lowest gastric pH and thereby minimize the extent of this potential interaction.

In this paper, we examined if the staggered dosing approach is appropriate by using modeling and simulation tools: the first was a retrospective analysis to assess the effect of H2RA use on dacomitinib exposure by using the dacomitinib PK data pooled from cancer patients treated with dacomitinib where an H2RA was allowed, and the second was a physiologically based pharmacokinetic (PBPK) modeling analysis to evaluate if changes in gastric pH by the staggered H2RA co-administration approach would minimize the impact of an H2RA on dacomitinib absorption.

## 2. Materials and Methods

### 2.1. Analysis of Clinical PK of Dacomitinib with and without H2RA Co-Administration

#### 2.1.1. Summary of Clinical Studies

This analysis included data from 11 clinical studies. An overview of the clinical studies is listed in Table 1. Patients in these studies who received continuous oral dosing schedule of dacomitinib at dose levels of 45 mg once daily (QD), 30 mg QD, 15 mg QD and 60 mg QD, with a cycle duration of 28 days, are included in the analysis dataset. All trials were conducted in accordance with the International Council on Harmonization Good Clinical Practice guidelines and the provisions of the Declaration of Helsinki. The institutional review board or independent ethics committee at each participating center approved the protocol. All patients provided written informed consent before enrollment.

#### 2.1.2. Dacomitinib PK Population

Patients in the 11 studies who received continuous oral dosing schedule of dacomitinib at dose levels of 45 mg once daily (QD), 30 mg QD, 15 mg QD and 60 mg QD, with a cycle duration of 28 days, are included in the analysis dataset. Plasma concentrations of dacomitinib and its active metabolite (PF-05199265) were collected and analyzed by liquid chromatography–tandem mass spectrometry as previously described [23,24]. PF-05199265 has been identified to have an inhibitory and selectivity profile similar to the parent molecule [24]. For each patient, steady-state trough concentrations (C_trough,ss_) were defined as an observed concentration collected at the nominal predose (0 h) time point on Day 1 of Cycle 2 through Cycle 10 with at least 14 days of continuous dacomitinib dosing at one dose level. C_trough,ss_ were dose normalized to 45 mg, calculated as C_trough,ss_ times 45 mg/dose for the analysis and reporting. Active moiety is the sum of exposures of dacomitinib and its active metabolite. C_trough,ss_ of total active drug moiety (parent dacomitinib + metabolite PF-05199265) were also calculated as

$${(C}_{trough,ss\;dacomitinib}+C_{trough,ss\;PF\text{-}05199265}\times \frac{469.4\;(molecular\;weight\;of\;dacomitinib)}{455.9\;(molecular\;weight\;of\;PF\text{-}05199265)}).$$

The C_trough,ss_ values were used to represent steady-state exposure in patients following administration of 45 mg QD dacomitinib.

The C_trough,ss_ collected prior to any reported H2RA use or at least 14 days after the last reported date of H2RA use served as the dacomitinib without H2RA reference. Dacomitinib co-administered with H2RA as the comparator was defined as C_trough,ss_ collected after the use of any reported H2RA continuously for at least 3 days. The H2RAs reported in these studies included cimetidine, famotidine, nizatidine and ranitidine.

#### 2.1.3. Statistical Analysis

A linear mixed effects model was used to perform the within-patient comparison of C_trough,ss_ for the dacomitinib parent, metabolite and active drug moiety C_trough,ss_, respectively, between dacomitinib co-administered with H2RA and dacomitinib without H2RA, as described by:

$$log\;\left(y_{ijk}\right)=µ+\theta_{1}\cdot x_{1jk}+\theta_{2}\cdot TAFD+\eta_{J}+\varepsilon_{ijk}$$

where

- $y_{ijk}$ = dacomitinib parent, metabolite and active moiety C_trough,ss_ for the $i^{th}$ group (test), the $j^{th}$ patient and the $k^{th}$ within patient observation.
- µ = mean dacomitinib parent, metabolite and active moiety C_trough,ss_ (natural log scale) for the reference group.
- θ_1_ = H2RA effect as the mean dacomitinib parent, metabolite and active moiety C_trough,ss_ difference (natural log scale) between test ($x_{1i}$ = 1) and reference ($x_{1i}$ = 0).
- θ_2_ = time effect for the test dacomitinib parent, metabolite and active moiety C_trough,ss_.
- TAFD = time of observed dacomitinib parent, metabolite and active moiety C_trough,ss_ after first dose in hours.
- $\eta_{j}$ = inter-patient random effect.
- $\varepsilon_{ijk}$ = intra-patient random error.

The effect of H2RA use on dacomitinib and its metabolite exposure was estimated by adjusted least square means for test and reference groups, compared by estimating the ratio of adjusted geometric means (test/reference) and the 90% CI for the ratio.

R version 3.4.1 (R Foundation for Statistical Computing. Vienna, Austria) was used for all data manipulation, analysis steps (using the lme() function of the nlme package in R), graphics and table creation.

### 2.2. PBPK Modeling and Sensitivity Analysis of Dacomitinib PK

#### 2.2.1. PBPK Model Development

PBPK modeling was used to evaluate the effect of changes in gastric pH on dacomitinib absorption. With physicochemical properties (e.g., Log P, pKa, mean precipitation time) and solubility data at different pH for dacomitinib, a two-compartment model coupled to the ACAT model was built using Gastroplus^TM^ version 9.7 (Simulation Plus, Inc., Lancaster, CA, USA) with default fasted gut physiology. Input parameters are provided in Table 2. Disposition PK parameters for dacomitinib were derived by initially fitting the PK data after intravenous (IV) administration from a study where the absolute bioavailability of dacomitinib was estimated by comparing oral to intravenous administration of dacomitinib in healthy volunteers [25]. All input parameters were provided in Table 2.

The model that utilized the default physiological conditions of Gastroplus was used to simulate the plasma profiles for a 45 mg oral dose in healthy volunteers from the same study. Visual comparison of the predicted to the observed PK profiles resulted in a decision to modify the oral absorption model to capture the initial phase of dacomitinib absorption. The modified model was developed by optimizing the absorption scale factor (ASF) model based on the clinical PK data to reflect the prolonged T_max_ of the plasma profile since all the systemic PK parameters were fixed after fitting the IV profile. The optimization steps resulted in well-captured observed C_max_ and T_max_.

#### 2.2.2. PBPK Model Verification

The model was validated using the mean plasma profiles of dacomitinib under 3 different conditions or treatments (fasted, fed, and PPI treatment using rabeprazole 40 mg administered QD for 7 days) following oral administration of a 45 mg dose in a clinical study in healthy volunteers to evaluate the food effect and effect of PPIs [11]. In this study, PK samples were collected at the same nominal times of 0, 1, 2, 4, 6, 8, 12, 24, 48, 72, 96, 120, 144, 168, 192, 216 and 264 h post-dose. The mean plasma drug concentration–time profile of dacomitinib was obtained for each condition, namely fasted, fed and PPI treatment, and respective exposure parameters (C_max_ and AUC_inf_) were derived from Gastroplus. The simulation was conducted using Gastroplus with the default physiological conditions of fasted or fed and by setting the gastric pH in the model to 6.0. This allowed for the simulation of the situations corresponding to fasted, fed and PPI treatment, respectively.

#### 2.2.3. PBPK Model Simulations

The gastric pH in the PBPK model was manipulated to enable the evaluation of a range of pH values from 1.0 to 6.0 during pharmacokinetic (PK) simulations. Parameter sensitivity analyses (PSAs) were performed on the PBPK model to assess the impact of pH variations from 1.0 to 5.0, which mimicked the effect of H2RAs on the absorption of dacomitinib.

## 3. Results

### 3.1. Analysis of Clinical PK of Dacomitinib with and without H2RA Co-Administration

As shown in Table 3, across the 11 studies (1450 patients treated with dacomitinib), there were 1001 patients with available dacomitinib C_trough,ss_ meeting the criteria defined in Section 3.1, and 86 total patients reported use of an H2RA. The within-patient H2RA–dacomitinib C_trough,ss_ analysis population consisted of 16 total patients with 57 dacomitinib C_trough,ss_ in the reference or test group. Of these 16 patients, 12 had metabolite and active moiety concentrations in the analysis population with 46 metabolite and active moiety C_trough,ss_ in the reference or test group.

The geometric mean (geometric coefficient of variation (CV%)) for dacomitinib parent C_trough,ss_ (dose normalized to 45 mg) for the reference group was 57.56 ng/mL (8.4%) based on 35 observations (Table 4). The geometric mean (geometric CV%) for dacomitinib C_trough,ss_ (dose normalized to 45 mg) for the test group was 53.2 ng/mL (18.7%) based on 22 observations. The geometric mean (geometric CV%) C_trough,ss_ (dose normalized to 45 mg) for the dacomitinib metabolite and active moiety for the reference group based on 29 observations were 6.41 ng/mL (47.8%) and 66.45 ng/mL (7.7%), respectively. The geometric mean (geometric CV%) C_trough,ss_ (dose normalized to 45 mg) for the dacomitinib metabolite and active moiety for the test group based on 17 observations were 8.81 ng/mL (41.6%) and 72.23 ng/mL (5.7%), respectively.

**Figure 1:**
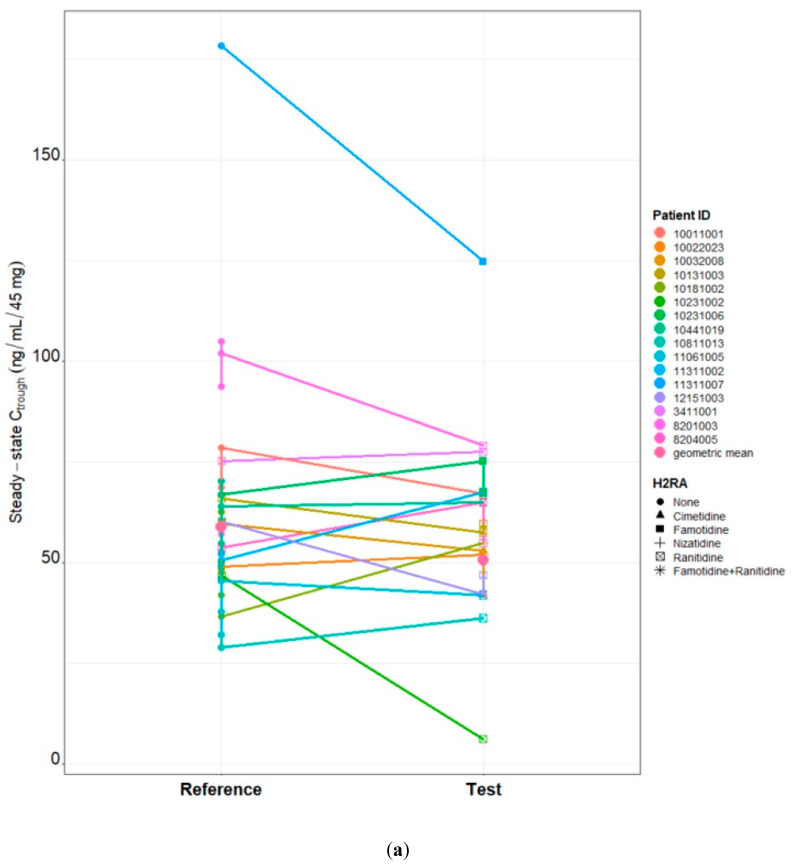

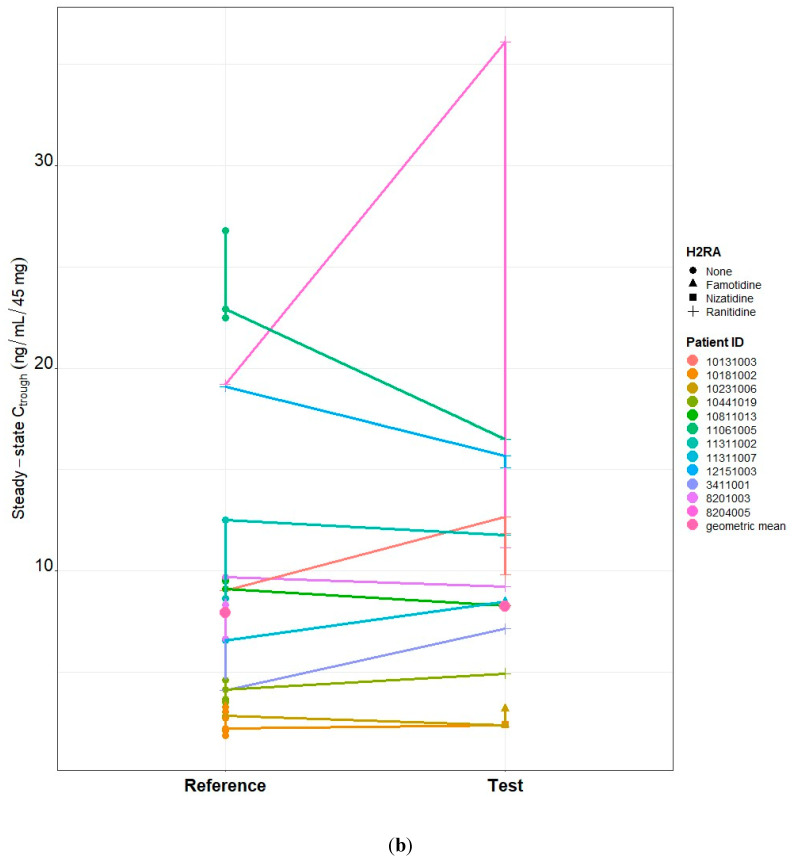

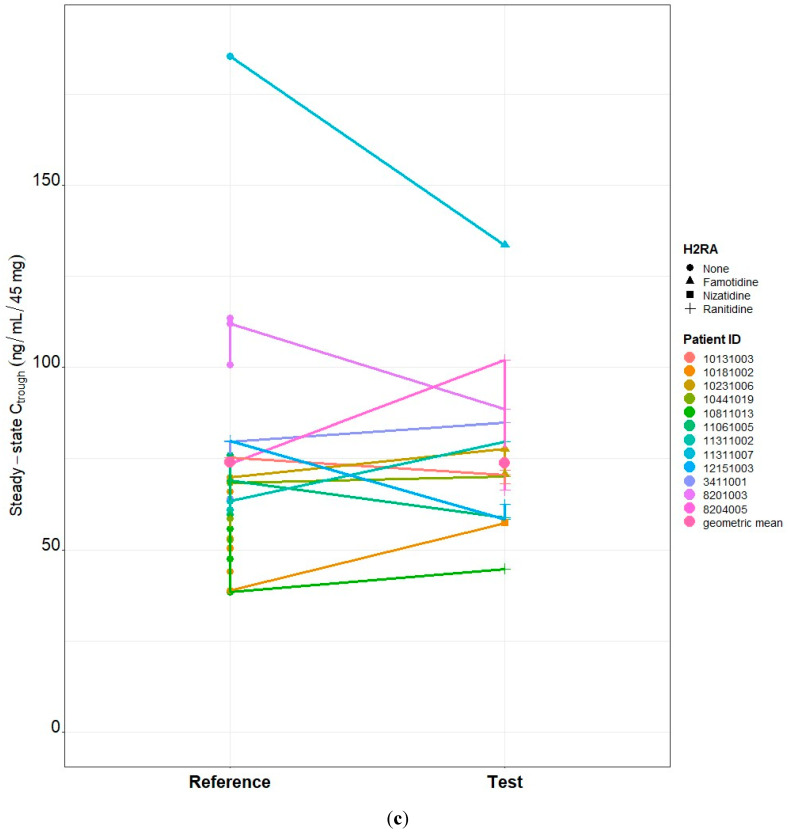
Dacomitinib parent, metabolite and active drug moiety C_trough,ss_ matchstick boxplot by patient and the H2RA medication used (disc: none; triangle: cimetidine; square: famotidine; cross: nizatidine; ballot box with x: ranitidine; sextile: famotidine + ranitidine). (**a**) Parent. (**b**) Metabolite. (**c**) Active drug moiety.

The effect of concomitant H2RA use (test) on dacomitinib parent, metabolite and active moiety C_trough,ss_ was evaluated by a linear mixed effects model and the test group was not statistically significant compared to the reference group (*p* = 0.1245, 0.6611 and 0.9668). Time was also evaluated in the model and was not significant (*p* = 0.6628, 0.9558 and 0.7304). The results of the statistical model showed that the geometric mean ratios (test/reference), expressed as a percentage, are 85.88% with a 90% CI of 72.9 to 101.1, 103.82% with a 90% CI of 89.9 to 119.8 and 99.77% with a 90% CI of 90.9 to 109.5, respectively. The results are consistent with the visual presentation of dacomitinib C_trough,ss_ reference and test values by patient (Figure 1).

### 3.2. PBPK Modeling and Sensitivity Analysis of Dacomitinib PK

**Figure 2:**
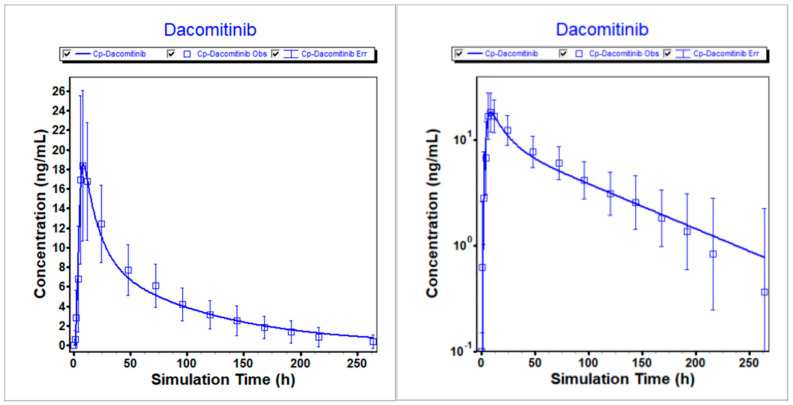
Simulated vs. observed PK profiles following 45 mg dacomitinib in fasted state. (**Left**) Linear scale. (**Right**) Logarithmic scale.

**Figure 3:**
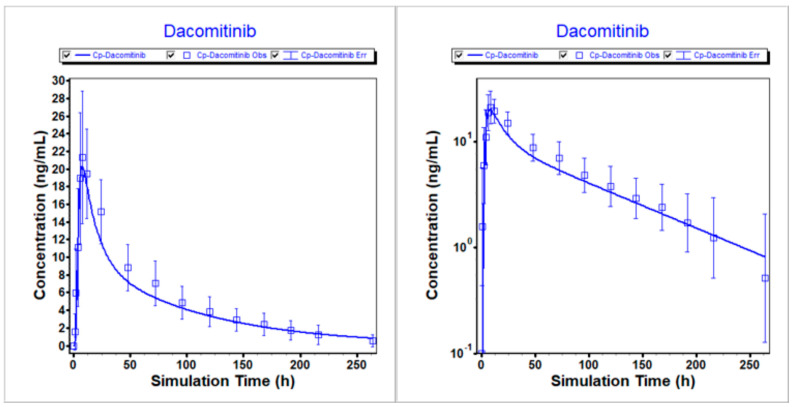
Simulated vs. observed PK profiles following 45 mg dacomitinib in fed state. (**Left**) Linear scale. (**Right**) Logarithmic scale.

**Figure 4:**
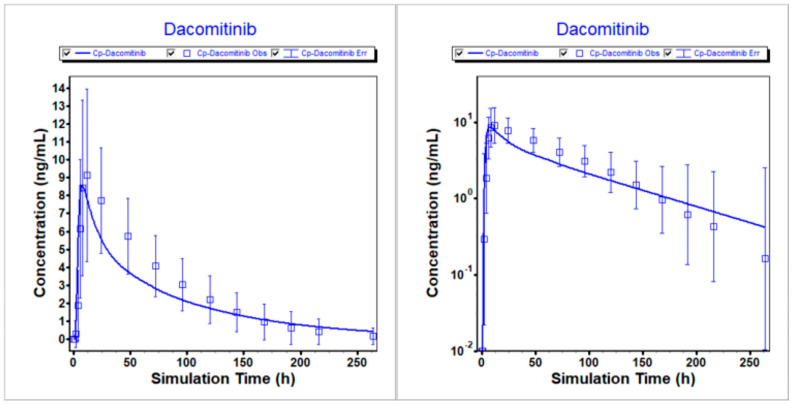
Simulated vs. observed PK profiles following 45 mg dacomitinib with PPI treatment. (**Left**) Linear scale. (**Right**) Logarithmic scale.

The PBPK model for dacomitinib was developed and optimized using in vitro and clinical data. Subsequently, the modified model was employed to predict the mean plasma profiles of dacomitinib after administering a 45 mg oral dose to healthy volunteers under different conditions, namely fasted, fed and PPI treatment. The comparison between the predicted and clinically observed PK results was summarized. This is demonstrated in Figure 2, Figure 3 and Figure 4, which clearly depict how the optimized model accurately describes the PK profiles of dacomitinib under varying conditions. Additionally, Table 5 presents the predicted-to-observed ratios for AUC_inf_ and C_max_, all of which fall within a twofold range of the observed values. Specifically, the ratios for AUC_inf_ range from 0.857 to 1.07, while those for C_max_ range from 0.940 to 1.01. In conclusion, these findings suggest that the PBPK model for dacomitinib has been established and can be used for estimating dacomitinib exposure (C_max_ and AUC_inf_) after oral administration under different situations based on the clinically observed PK data obtained from the clinical study of food effects and PPIs with dacomitinib [11].

**Figure 5:**
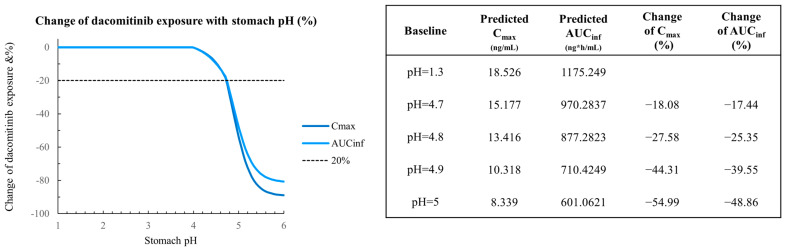
PSA on 45 mg dacomitinib AUC_inf_ and C_max_ for the pH range 1–5.

The validated model was then utilized to predict the impact of pH changes on the absorption of dacomitinib through PSA analysis. Figure 5 illustrates the PSA values for AUC_inf_ and C_max_ for dacomitinib at a dose of 45 mg within the pH range of 1–5 (pH range 1–2 represents normal stomach, while range 3–5 represents post-H2RA administration stomach) [27]. The results indicated no overt changes in AUC_inf_ and C_max_ for dacomitinib in the pH range of 1 to 4.7. However, at the extreme situation of pH 5, the predicted AUC_inf_ decreased by 48.86% and C_max_ decreased by 54.99%. These simulation results suggest that varying the stomach pH within the range of 1–5 does not appear to significantly affect the absorption of dacomitinib.

## 4. Discussion

Lung cancer is one of the five cancer types with the highest prevalence of ARA use for indications like gastroesophageal reflux disease (GERD), esophagitis or peptic ulcers [28]. Per AAFP guidelines, the first-line treatment for GERD is a trial of an H2RA for 8 weeks followed by switching to a PPI [29]. Due to the differences in the magnitude and duration of gastric pH elevation among various ARAs, the potential for pH-dependent DDIs can vary depending on the type, dosage and timing of administration of the ARA in relation to the affected medication. When an orally administered drug with pH-sensitive solubility is co-administered with an ARA, the absorption of the drug may be altered, leading to undesirable clinical consequences. In general, if a drug is determined to have the potential for a pH-dependent DDI, a clinical study should be conducted to characterize the effect of ARAs on the pharmacokinetics of the investigational drug [30]. Among the currently approved ARAs, PPIs result in prolonged effects on gastric pH and thus are commonly used as ARA probes in such clinical studies. The effects of other classes of ARAs are not as well characterized; however, it is important to gain better understanding and, ultimately, dosing recommendations for investigational drugs co-administered with all classes of ARAs.

Dacomitinib is a weak base with a pH-dependent solubility profile over the range found in the human gastrointestinal tract. A dedicated clinical pharmacology study in healthy volunteers [11] demonstrated that co-administration of dacomitinib with multiple doses of rabeprazole (a PPI) decreased the dacomitinib AUC by 39%, while a pilot study in patients showed that co-administration of dacomitinib with a local antacid (Maalox^®^ Maximum Strength, 400 mg/5 mL) did not cause clinically relevant changes in dacomitinib concentrations [2]. The effect of an H2RA on dacomitinib’s PKs has not been examined in any clinical studies and yet is expected to be less than that of a PPI.

Existing patient data from clinical studies offer an opportunity to characterize the landscape of concomitant H2RA use as well as to answer the question of potential PK effects as a result of these pH-dependent DDIs in the patient population. Not only is this analysis more clinically relevant, but within-patient comparisons can reduce the bias caused by nonrandomized retrospective analyses. In this retrospective analysis, a within-patient comparison of the dacomitinib C_trough,ss_ of parent, metabolite and active moiety between dacomitinib without concomitant H2RA use and dacomitinib co-administered with an H2RA medication was performed to evaluate the effect of H2RA use on the dacomitinib PKs. The within-patient analysis allows the comparison with smaller sample size and minimized the random noise. A total of 1450 patients from 11 prospectively planned clinical studies of dacomitinib were included, with >1000 reported C_trough,ss_ values of dacomitinib. Of this large database, 16 patients of the C_trough,ss_ analysis population met the criteria to evaluate the test comparison for the effect of H2RAs on dacomitinib exposure. These 16 patients had (1) at least one C_trough,ss_ value measured before the use of any reported H2RAs (including four commonly used H2RAs: famotidine, ranitidine, cimetidine or nizatidine) or at least 14 days after the last reported H2RA use and (2) at least 1 C_trough,ss_ value measured after any reported H2RA use for at least 3 consecutive days. Of these 16 patients, 12 had metabolite and active moiety concentrations in the analysis population. By using a within-patient comparison (each patient serves as his or her own control), fewer patients are needed for a precise evaluation of the hypothesis. This was borne out by the reported narrow confidence limits (as shown in Table 4) for the geometric mean ratios of C_trough,ss_ for dacomitinib as well as its active metabolite and total active drug moiety, with and without concomitant H2RAs. The methods used provide reasonable estimates of the effect with 16 patients.

PBPK modeling has become increasingly popular among drug developers to address the DDI effects of ARAs. This modeling approach establishes a direct connection between PK and gastrointestinal physiological parameters as well as compound and formulation properties [31,32,33,34]. To predict the effect of H2RAs on the oral absorption of dacomitinib, we developed an oral absorption model using PBPK. This model was constructed and validated using in vitro and clinical data from a DDI study involving rabeprazole 40 mg and dacomitinib PKs [11], as well as an absolute bioavailability study comparing oral and intravenous administration in healthy volunteers [25]. The model demonstrated reasonably accurate predictions of the mean plasma concentration of dacomitinib at a 45 mg dose in healthy volunteers, consistent with the observations from the aforementioned clinical trials.

After the verification step, the model was utilized to depict the PK profiles of dacomitinib under different scenarios, including fasted, fed state and in combination with a PPI. The resulting PK profiles from these simulations were compared to the observed profiles from the DDI study with rabeprazole 40 mg and dacomitinib PKs [11]. The predicted-to-observed ratios fell within the ranges of 0.857–1.07 for AUC_inf_ and 0.940–1.01 for C_max_. These findings indicate that the PBPK model adequately describes the pharmacokinetic exposure (C_max_ and AUC_inf_) of dacomitinib and demonstrates good accuracy.

Sensitivity analysis was conducted through varying gastric pH to explore its impact on dacomitinib exposures. The results revealed that the exposures of dacomitinib did not significantly change when the gastric pH varied from 1 to 4.8. However, under extreme circumstances where the pH reached 5 (which is unlikely or transient after dosing with H2RAs) [35,36,37], the predicted AUC_inf_ exhibited a decrease of 48.86%, while the predicted C_max_ decreased by 54.99%.

Similar to PPIs, H2RAs cause a substantial reduction in acid secretion by reversibly inhibiting the histamine (H2) receptor on the acid-secreting parietal cell of the stomach. Unlike the prolonged effect of PPIs, however, H2RAs do not completely and irreversibly inhibit acid production, resulting in short-lived inhibition of acid secretion [38]. The onset of inhibition occurs after approximately 4 h with maximal inhibition after about 8 h, and acid secretion returns after about 12 h [39]. As a result, compared to PPIs, H2RAs generally have a lower degree of reduced bioavailability and systemic exposure when used with oral kinase inhibitors, which is consistent with their weaker efficacy [40,41,42,43,44]. Therefore, the impact of H2RAs on survival outcomes in patients is expected to be less profound than the PPI impact, especially considering the recommended scheduling of H2RA administration several hours apart from dacomitinib [2].

Retrospective analyses of survival data from clinical trials in patients with lung cancer indicates that ARA therapy may not have any impact on efficacy outcomes. Li J et al. have reported that even with a potential 37% decrease in exposure after co-administration of PPIs, there was no difference in efficacy in patients with EGFR-positive NSCLC as evidenced by PFS and OS [45]. Similar findings have been reported for other TKIs. In the phase III study of 485 NSCLC patients receiving erlotinib as second- or third-line therapy, no significant differences in PFS or OS were observed with ARA therapy (either PPI or H2RA therapy) [46,47]. Van De Sijpe, G. et al. have concluded there was no evidence of a negative impact on outcome in mRCC patients treated with first-line pazopanib when co-administered with PPIs/H2RAs. Measurements of PFS, OS and tumor response did not show any significant effects in the patient series [48,49,50].

Our research has some limitations. Firstly, for the linear mixed effects model analysis pooling 11 clinical studies retrospectively, patients were treated as outpatients and were considered to be taking an H2RA when this was mentioned in the electronic medical record. However, we do not have information about the dosing administration and dose of H2RA medication. This lack of data regarding the specific timing and dosage of H2RA administration is important because different H2RAs and doses can vary in their ability to suppress stomach acidity. In terms of PBPK modeling, in terms of simulation of the pH effect, we kept the gastric pH constant in our predictions, which is a common practice in most PBPK models when predicting pH-dependent DDIs [35,51]. However, it has been shown in numerous studies that the gastric pH undergoes dynamic changes after administration of ARAs [36,37,52]. Therefore, incorporating a dynamic pH change during simulation could improve the accuracy of our model in predicting the impact of gastric pH changes. Therefore, incorporating a dynamic pH change during the simulation could improve the accuracy of our model in predicting the impact of gastric pH change. In addition, in our study, we assume that H2RAs only changed the gastric pH and not the other parameters. However, certain studies have suggested that H2RAs may also delay gastric emptying, which could contribute to changes in drug absorption [53,54]. Additionally, Dong Z’s study on the application of PBPK models to predict gastric pH-dependent DDIs for weak-base drugs identified gaps in correctly predicting changes in AUC or C_max_ in the presence of ARAs [27]. This suggests that there is room for further refinement of our PBPK model as more data become available. While our research provides valuable insights, it is important to acknowledge these limitations and consider future improvements to enhance the accuracy and applicability of our findings [42].

Overall, the results from the retrospective analysis using within-patient comparison of clinical PK data and the PBPK modeling indicated that the concurrent use of H2RAs unlikely impacts dacomitinib’s PKs and subsequently does not impact clinical benefits in patients.

## 5. Conclusions

Based on in vitro, in silico and retrospective analysis of pooled data from clinical trials, co-administered H2RAs do not show any statistical or clinically meaningful pH-dependent DDIs. Together, we provided the rationales supporting a staggered dosing strategy of dacomitinib administration with an H2RA as indicated on the label.

## Figures and Tables

**Table 1:** Overview of clinical studies.

Study	Study Description	Reference
1	A phase 1, open-label, dose-escalation study to evaluate safety, PKs and PD of 2 dosing schedules of dacomitinib in patients with advanced malignant solid tumors	[12]
2	A phase 2, open-label, two-arm study to evaluate the efficacy of dacomitinib in patients with advanced NSCLC after failure of at least one prior chemotherapy regimen and failure of prior treatment with erlotinib	[13]
3	A phase 1/2, open-label, single-arm study to determine the recommended phase 2 dose and evaluate the efficacy of dacomitinib in patients in Korea with Kras wild-type advanced NSCLC, which is refractory to chemotherapy and erlotinib or gefitinib	[14]
4	A clinical phase 1 study of dacomitinib in patients with advanced malignant solid tumors	[15]
5	A randomized, double-blind phase 3 efficacy and safety study of dacomitinib vs. erlotinib for the treatment of advanced NSCLC following progression after, or intolerance to, at least one prior chemotherapy	[16]
6	A phase 1, open-label, single-arm study to evaluate the effect of dacomitinib on the PKs of dextromethorphan in patients with advanced malignant solid tumors	[17]
7	A phase 2, open-label study of dacomitinib in selected patients with advanced adenocarcinoma of the lung	[18]
8	Clinical phase 2 multicenter study of dacomitinib in patients with recurrent or metastatic squamous cell carcinoma of the head and neck	[19]
9	A randomized, phase 2 study of dacomitinib versus erlotinib for the treatment of advanced NSCLC after failure of at least 1 prior chemotherapy regimen	[20]
10	A phase 2 study of dacomitinib in advanced NSCLC (post-chemotherapy or select first-line patients) to evaluate prophylactic intervention on dermatologic and gastrointestinal adverse events and patient-reported outcomes	[21]
11	A randomized, open-label, phase 3 efficacy and safety study of dacomitinib versus gefitinib for the first-line treatment of locally advanced or metastatic NSCLC in subjects with EGFR-activating mutation(s)	[22]

NSCLC = non-small-cell lung cancer; EGFR = epidermal growth factor receptor; PKs = pharmacokinetics; PD = pharmacodynamics.

**Table 2:** Key physicochemical, PK inputs and ASF model used in the models.

Parameter	Dacomitinib Value	Source
Molecular weight (g/mol)	469.9	[26]
LogP	5.3 (neutral), 1.8 (cationic)	[26]
pKa	5.03 (base)	[26]
Solubility (mg/mL)	10.0 at pH 1.28 3.7 at pH 4.71 0.34 at pH 5.09 0.23 at pH 5.17 0.16 at pH 5.3 0.006 at pH 6.13 0.001 at pH 6.94	[26]
Effective human permeability (cm/s)	1.18 × 10^4^	[26]
Permeability source	Caco-2	[26]
Precipitation time (s)	900	Default value in GastroPlus™ 9.7
Particle size radius (μm)	58	[26]
Disposition model parameters		
CL (L/h)	36.1	Automatic estimation using PK Plus based on IV data from clinical study [25]
Vc (L/kg)	21.9	
V2 (L/kg)	19.018	
K12 (1/h)	0.033	
K21 (1/h)	0.038	
ASF model (Modified)		
Stomach	0	Default value in GastroPlus™ 9.7
Duodenum	0.020	Optimized value (initial value from default value in G+: 2.695)
Jejunum	0.081	Optimized value (initial value from default value in G+: 2.646)
Jejunum	0.152	Optimized value (initial value from default value in G+: 2.649)
Ileum	0.029	Optimized value (initial value from default value in G+: 2.603)
Ileum	10.00	Optimized value (initial value from default value in G+: 2.582)
Ileum	40.00	Optimized value (initial value from default value in G+: 2.512)
Caecum	120.00	Optimized value (initial value from default value in G+: 11.49)
Asc Colon	20.00	Optimized value (initial value from default value in G+: 25.14)

**Table 3:** Analysis populations by study.

Study ID	Study Number											Total
	1	2	3	4	5	6	7	8	9	10	11	
Patients treated with dacomitinib	121	66	55	13	436	15	119	69	93	236	227	1450
Patients with at least one dacomitinib C_trough,ss_	20	50	48	11	311	6	110	57	60	125	203	1001
H2RA use population	1	3	5	0	40	0	4	4	2	13	14	86
H2RA–Dacomitinib C_trough,ss_ analysis population	0	0	3	0	6	0	1	0	0	3	3	16
H2RA–metabolite and active moiety C_trough,ss_ analysis population	0	0	0	0	6	0	0	0	0	3	3	12

**Table 4:** Statistical comparison of dacomitinib parent, metabolite and active drug moiety C_trough,ss_.

Parameter	Geometric Mean (ng/mL) (Geometric CV%)		Geometric Mean Ratio (%) (90% CI)
	Reference	Test	
Dacomitinib C_trough,ss_	57.56 (8.4)	53.2 (18.7)	85.88 (72.9, 101.11)
Metabolite C_trough,ss_	6.41 (47.8)	8.81 (41.6)	103.82 (89.9–119.8)
Active drug moiety C_trough,ss_	66.45 (7.7)	72.23 (5.7)	99.77 (90.9–109.5)
CI: confidence interval			

**Table 5:** Comparison of simulated and observed exposures following 45 mg dacomitinib under different situations.

	C_max_ (ng/mL)		AUC_inf_ (ng·h/mL)		Ratio of C_max_	Ratio of AUC_inf_
	Predicted	Observed	Predicted	Observed		
Fasted state	18.526	18.41	1175.2	1171	1.01	1.00
Fed state	20.317	21.35	1246.3	1163	0.952	1.07
Co-administration with PPI	8.576	9.125	613.23	715.96	0.940	0.857

## Data Availability

Upon request, and subject to review, Pfizer will provide the data that support the findings of this analysis. Subject to certain criteria, conditions and exceptions, Pfizer may also provide access to the related individual de-identified participant data. See https://www.pfizer.com/science/clinical-trials/data-and-results (accessed on 6 November 2023) for more information.

## References

[1] Shirley M. (2018). Dacomitinib: First Global Approval. Drugs.

[2] US Food and Drug Administration (FDA) VIZIMPRO^®^ (Dacomitinib) Prescribing Information. https://www.accessdata.fda.gov/drugsatfda_docs/label/2018/211288s000lbl.pdf.

[3] Sullivan I., Planchard D. (2016). Next-Generation EGFR Tyrosine Kinase Inhibitors for Treating EGFR-Mutant Lung Cancer beyond First Line. Front. Med..

[4] Mok T.S., Cheng Y., Zhou X., Lee K.H., Nakagawa K., Niho S., Chawla A., Rosell R., Corral J., Migliorino M.R. (2021). Updated Overall Survival in a Randomized Study Comparing Dacomitinib with Gefitinib as First-Line Treatment in Patients with Advanced Non-Small-Cell Lung Cancer and EGFR-Activating Mutations. Drugs.

[5] Wu Y.L., Cheng Y., Zhou X., Lee K.H., Nakagawa K., Niho S., Tsuji F., Linke R., Rosell R., Corral J. (2017). Dacomitinib versus gefitinib as first-line treatment for patients with EGFR-mutation-positive non-small-cell lung cancer (ARCHER 1050): A randomised, open-label, phase 3 trial. Lancet Oncol..

[6] European Medicines Agency (EMA) VIZIMPRO^®^ (Dacomitinib) Summary of Product characteristics (SmPC). https://www.ema.europa.eu/en/documents/product-information/vizimpro-epar-product-information_en.pdf.

[7] Budha N.R., Benet L.Z., Ware J.A. (2013). Response to “Drug interactions produced by proton pump inhibitors: Not simply a pH effect”. Clin. Pharmacol. Ther..

[8] Numico G., Fusco V., Franco P., Roila F. (2017). Proton Pump Inhibitors in cancer patients: How useful they are? A review of the most common indications for their use. Crit. Rev. Oncol. Hematol..

[9] Ruiz-Garcia A., Tan W., Li J., Haughey M., Masters J., Hibma J., Lin S. (2020). Pharmacokinetic Models to Characterize the Absorption Phase and the Influence of a Proton Pump Inhibitor on the Overall Exposure of Dacomitinib. Pharmaceutics.

[10] Janne P.A., Boss D.S., Camidge D.R., Britten C.D., Engelman J.A., Garon E.B., Guo F., Wong S., Liang J., Letrent S. (2011). Phase I dose-escalation study of the pan-HER inhibitor, PF299804, in patients with advanced malignant solid tumors. Clin. Cancer Res..

[11] Ruiz-Garcia A., Masters J.C., Mendes da Costa L., LaBadie R.R., Liang Y., Ni G., Ellery C.A., Boutros T., Goldberg Z., Bello C.L. (2016). Effect of food or proton pump inhibitor treatment on the bioavailability of dacomitinib in healthy volunteers. J. Clin. Pharmacol..

[12] Study to Evaluate the Safety, Pharmacokinetics, and Pharmacodynamics of PF-00299804 in Patients with Advanced Solid Tumors. Clinicaltrials.Gov Identifier: NCT0022512. https://clinicaltrials.gov/study/NCT00225121?term=NCT00225121&rank=1.

[13] Reckamp K.L., Giaccone G., Camidge D.R., Gadgeel S.M., Khuri F.R., Engelman J.A., Koczywas M., Rajan A., Campbell A.K., Gernhardt D. (2014). A phase 2 trial of dacomitinib (PF-00299804), an oral, irreversible pan-HER (human epidermal growth factor receptor) inhibitor, in patients with advanced non-small cell lung cancer after failure of prior chemotherapy and erlotinib. Cancer.

[14] Park K., Cho B.C., Kim D.W., Ahn M.J., Lee S.Y., Gernhardt D., Taylor I., Campbell A.K., Zhang H., Giri N. (2014). Safety and efficacy of dacomitinib in korean patients with KRAS wild-type advanced non-small-cell lung cancer refractory to chemotherapy and erlotinib or gefitinib: A phase I/II trial. J. Thorac. Oncol..

[15] Takahashi T., Boku N., Murakami H., Naito T., Tsuya A., Nakamura Y., Ono A., Machida N., Yamazaki K., Watanabe J. (2012). Phase I and pharmacokinetic study of dacomitinib (PF-00299804), an oral irreversible, small molecule inhibitor of human epidermal growth factor receptor-1, -2, and -4 tyrosine kinases, in Japanese patients with advanced solid tumors. Investig. New Drugs.

[16] Ramalingam S.S., O’Byrne K., Boyer M., Mok T., Janne P.A., Zhang H., Liang J., Taylor I., Sbar E.I., Paz-Ares L. (2016). Dacomitinib versus erlotinib in patients with EGFR-mutated advanced nonsmall-cell lung cancer (NSCLC): Pooled subset analyses from two randomized trials. Ann. Oncol..

[17] A Study to Test the Impact of PF-00299804 on How the Body Handles Dextromethorphan in Cancer Patients. Clinicaltrials.Gov Identifier: NCT00728468. NCT00728468.

[18] Kris M.G., Camidge D.R., Giaccone G., Hida T., Li B.T., O’Connell J., Taylor I., Zhang H., Arcila M.E., Goldberg Z. (2015). Targeting HER2 aberrations as actionable drivers in lung cancers: Phase II trial of the pan-HER tyrosine kinase inhibitor dacomitinib in patients with HER2-mutant or amplified tumors. Ann. Oncol..

[19] Kim H.S., Kwon H.J., Jung I., Yun M.R., Ahn M.J., Kang B.W., Sun J.M., Kim S.B., Yoon D.H., Park K.U. (2015). Phase II clinical and exploratory biomarker study of dacomitinib in patients with recurrent and/or metastatic squamous cell carcinoma of head and neck. Clin. Cancer Res..

[20] Ramalingam S.S., Blackhall F., Krzakowski M., Barrios C.H., Park K., Bover I., Seog Heo D., Rosell R., Talbot D.C., Frank R. (2012). Randomized phase II study of dacomitinib (PF-00299804), an irreversible pan-human epidermal growth factor receptor inhibitor, versus erlotinib in patients with advanced non-small-cell lung cancer. J. Clin. Oncol..

[21] Lacouture M.E., Keefe D.M., Sonis S., Jatoi A., Gernhardt D., Wang T., Doherty J.P., Giri N., Nadanaciva S., O’Connell J. (2016). A phase II study (ARCHER 1042) to evaluate prophylactic treatment of dacomitinib-induced dermatologic and gastrointestinal adverse events in advanced non-small-cell lung cancer. Ann. Oncol..

[22] Cheng Y., Mok T.S., Zhou X., Lu S., Zhou Q., Zhou J., Du Y., Yu P., Liu X., Hu C. (2021). Safety and efficacy of first-line dacomitinib in Asian patients with EGFR mutation-positive non-small cell lung cancer: Results from a randomized, open-label, phase 3 trial (ARCHER 1050). Lung Cancer.

[23] Bello C.L., Smith E., Ruiz-Garcia A., Ni G., Alvey C., Loi C.M. (2013). A phase I, open-label, mass balance study of [(14)C] dacomitinib (PF-00299804) in healthy male volunteers. Cancer Chemother. Pharmacol..

[24] Piscitelli J., Chen J., LaBadie R.R., Salageanu J., Chung C.H., Tan W. (2022). The Effect of Hepatic Impairment on the Pharmacokinetics of Dacomitinib. Clin. Drug Investig..

[25] A Study to Assess Absorption of Study Drug Dacomitinib (PF-00299804), Given as an Oral Tablet Compared to an Intravenous Infusion in Healthy Volunteers. Clinicaltrials.Gov Identifier: NCT01796327. NCT01796327.

[26] US Food and Drug Administration (FDA) VIZIMPRO^®^ (Dacomitinib) Review. https://www.accessdata.fda.gov/drugsatfda_docs/nda/2018/211288Orig1s000ChemR.pdf.

[27] Dong Z., Li J., Wu F., Zhao P., Lee S.C., Zhang L., Seo P., Zhang L. (2020). Application of Physiologically-Based Pharmacokinetic Modeling to Predict Gastric pH-Dependent Drug-Drug Interactions for Weak Base Drugs. CPT Pharmacomet. Syst. Pharmacol..

[28] Smelick G.S., Heffron T.P., Chu L., Dean B., West D.A., Duvall S.L., Lum B.L., Budha N., Holden S.N., Benet L.Z. (2013). Prevalence of acid-reducing agents (ARA) in cancer populations and ARA drug-drug interaction potential for molecular targeted agents in clinical development. Mol. Pharm..

[29] DeVault K.R., Castell D.O. (1995). Guidelines for the diagnosis and treatment of gastroesophageal reflux disease. Practice Parameters Committee of the American College of Gastroenterology. Arch. Intern. Med..

[30] US Food and Drug Administration (FDA) Guidance “Evaluation of Gastric pH-Dependent Drug Interactions with Acid-Reducing Agents: Study Design, Data Analysis, and Clinical Implications”. https://www.fda.gov/regulatory-information/search-fda-guidance-documents/evaluation-gastric-ph-dependent-drug-interactions-acid-reducing-agents-study-design-data-analysis.

[31] Wagner C., Pan Y., Hsu V., Grillo J.A., Zhang L., Reynolds K.S., Sinha V., Zhao P. (2015). Predicting the effect of cytochrome P450 inhibitors on substrate drugs: Analysis of physiologically based pharmacokinetic modeling submissions to the US Food and Drug Administration. Clin. Pharmacokinet..

[32] Wagner C., Pan Y., Hsu V., Sinha V., Zhao P. (2016). Predicting the Effect of CYP3A Inducers on the Pharmacokinetics of Substrate Drugs Using Physiologically Based Pharmacokinetic (PBPK) Modeling: An Analysis of PBPK Submissions to the US FDA. Clin. Pharmacokinet..

[33] Dodd S., Kollipara S., Sanchez-Felix M., Kim H., Meng Q., Beato S., Heimbach T. (2019). Prediction of ARA/PPI Drug-Drug Interactions at the Drug Discovery and Development Interface. J. Pharm. Sci..

[34] Mitra A., Kesisoglou F., Beauchamp M., Zhu W., Chiti F., Wu Y. (2011). Using absorption simulation and gastric pH modulated dog model for formulation development to overcome achlorhydria effect. Mol. Pharm..

[35] Parrott N.J., Yu L.J., Takano R., Nakamura M., Morcos P.N. (2016). Physiologically Based Absorption Modeling to Explore the Impact of Food and Gastric pH Changes on the Pharmacokinetics of Alectinib. AAPS J..

[36] Walt R.P., Gomes M.D., Wood E.C., Logan L.H., Pounder R.E. (1983). Effect of daily oral omeprazole on 24 hour intragastric acidity. Br. Med. J..

[37] Tolman K.G., Sanders S.W., Buchi K.N., Karol M.D., Jennings D.E., Ringham G.L. (1997). The effects of oral doses of lansoprazole and omeprazole on gastric pH. J. Clin. Gastroenterol..

[38] Wolfe M.M., Soll A.H. (1988). The physiology of gastric acid secretion. N. Engl. J. Med..

[39] Sachs G., Shin J.M., Hunt R. (2010). Novel approaches to inhibition of gastric acid secretion. Curr. Gastroenterol. Rep..

[40] US Food and Drug Administration (FDA) Bosulif^®^ (Bosutinib) Prescribing Informatio. https://www.accessdata.fda.gov/drugsatfda_docs/label/2020/203341s018lbl.pdf.

[41] US Food and Drug Administration (FDA) Sprycel^®^ (Dasatinib) Prescribing Information. https://www.accessdata.fda.gov/drugsatfda_docs/label/2021/021986s025lbl.pdf.

[42] US Food and Drug Administration (FDA) Tarceva^®^ (Erlotinib) Prescribing Information. https://www.accessdata.fda.gov/drugsatfda_docs/label/2010/021743s14s16lbl.pdf.

[43] US Food and Drug Administration (FDA) IBRANCE^®^ (Palbociclib) Prescribing Information. https://www.accessdata.fda.gov/drugsatfda_docs/label/2017/207103s004lbl.pdf.

[44] DeVault K.R., Castell D.O., Practice Parameters Committee of the American College of Gastroenterology (2005). Updated guidelines for the diagnosis and treatment of gastroesophageal reflux disease. Am. J. Gastroenterol..

[45] Li J., Nickens D., Wilner K., Tan W. (2021). Evaluation of the Effect of Proton Pump Inhibitors on the Efficacy of Dacomitinib and Gefitinib in Patients with Advanced Non-Small Cell Lung Cancer and EGFR-Activating Mutations. Oncol. Ther..

[46] Rosell R., Carcereny E., Gervais R., Vergnenegre A., Massuti B., Felip E., Palmero R., Garcia-Gomez R., Pallares C., Sanchez J.M. (2012). Erlotinib versus standard chemotherapy as first-line treatment for European patients with advanced EGFR mutation-positive non-small-cell lung cancer (EURTAC): A multicentre, open-label, randomised phase 3 trial. Lancet Oncol..

[47] Hilton J.F., Tu D., Seymour L., Shepherd F.A., Bradbury P.A. (2013). An evaluation of the possible interaction of gastric acid suppressing medication and the EGFR tyrosine kinase inhibitor erlotinib. Lung Cancer.

[48] Van De Sijpe G., Beuselinck B., Van Nieuwenhuyse T., Poncelet R., Bechter O., Albersen M., Roussel E., Baldewijns M., Tack J., Spriet I. (2020). Impact of concomitant acid suppressive therapy on pazopanib efficacy and dose reductions in patients with metastatic renal cell carcinoma. Eur. J. Clin. Pharmacol..

[49] McAlister R.K., Aston J., Pollack M., Du L., Koyama T., Chism D.D. (2018). Effect of Concomitant pH-Elevating Medications with Pazopanib on Progression-Free Survival and Overall Survival in Patients with Metastatic Renal Cell Carcinoma. Oncologist.

[50] Mir O., Touati N., Lia M., Litiere S., Le Cesne A., Sleijfer S., Blay J.Y., Leahy M., Young R., Mathijssen R.H.J. (2019). Impact of Concomitant Administration of Gastric Acid-Suppressive Agents and Pazopanib on Outcomes in Soft-Tissue Sarcoma Patients Treated within the EORTC 62043/62072 Trials. Clin. Cancer Res..

[51] Cristofoletti R., Patel N., Dressman J.B. (2017). Assessment of Bioequivalence of Weak Base Formulations Under Various Dosing Conditions Using Physiologically Based Pharmacokinetic Simulations in Virtual Populations. Case Examples: Ketoconazole and Posaconazole. J. Pharm. Sci..

[52] Bruley des Varannes S., Levy P., Lartigue S., Dellatolas F., Lemaire M., Galmiche J.P. (1994). Comparison of lansoprazole with omeprazole on 24-hour intragastric pH, acid secretion and serum gastrin in healthy volunteers. Aliment. Pharmacol. Ther..

[53] Rasmussen L., Oster-Jorgensen E., Qvist N., Pedersen S.A. (1999). The effects of omeprazole on intragastric pH, intestinal motility, and gastric emptying rate. Scand. J. Gastroenterol..

[54] Parkman H.P., Urbain J.L., Knight L.C., Brown K.L., Trate D.M., Miller M.A., Maurer A.H., Fisher R.S. (1998). Effect of gastric acid suppressants on human gastric motility. Gut.

